# Du-Huo-Ji-Sheng-Tang Attenuates Inflammation of TNF-Tg Mice Related to Promoting Lymphatic Drainage Function

**DOI:** 10.1155/2016/7067691

**Published:** 2016-04-27

**Authors:** Yan Chen, Jinlong Li, Qiang Li, Tengteng Wang, Lianping Xing, Hao Xu, Yongjun Wang, Qi Shi, Quan Zhou, Qianqian Liang

**Affiliations:** ^1^Department of Orthopaedics, Longhua Hospital, Shanghai University of Traditional Chinese Medicine, Shanghai 200032, China; ^2^Department of Pathology and Laboratory Medicine, University of Rochester Medical Center, 601 Elmwood Avenue, Rochester, NY 14642, USA; ^3^Center for Musculoskeletal Research, University of Rochester Medical Center, 601 Elmwood Avenue, Rochester, NY 14642, USA

## Abstract

To investigate whether Du-Huo-Ji-Sheng-Tang (DHJST) attenuate inflammation of RA related to lymphatic drainage function in vivo, we treated eight 3-month-old TNF-Tg mice with DHJST (12 g/kg) or the same volume of physiological saline once every day for 12 weeks, and 3-month-old WT littermates were used as negative control. After twelve weeks, we performed NIR-ICG imaging and found that DHJST increased the ICG clearance at the footpad and the pulse of efferent lymphatic vessel between popliteal lymph node and footpad. Histology staining at ankle joints showed that DHJST decreases synovial inflammation, bone erosion, cartilage erosion, and TRAP+ osteoclast area in TNF-Tg mice. Immunohistochemical staining by using anti-Lyve-1 and anti-podoplanin antibody showed that DHJST stimulated lymphangiogenesis in ankle joints of TNF-Tg mice. And zebrafish study suggested that DHJST promoted the formation of lymphatic thoracic duct. In conclusion, DHJST inhibits inflammation severity and promotes lymphangiogenesis and lymphatic drainage function of TNF-Tg mice.

## 1. Introduction

Rheumatoid arthritis (RA) is a chronic autoimmune disease, characterized by progressive destruction of cartilage and bone, infiltration of inflammatory cells in joints, and presence of proliferative synovitis, usually compromising both the quality and duration of life. The pathology of this disease is complex, including the activation and infiltration of various populations of immune cells, which release inflammatory mediators into the synovial membrane of affected joints. The lymphatic system has vital functions in draining interstitial fluid from tissues into the blood and immune surveillance. Several clinical and laboratory observations indicate that inflammatory arthritis is associated with changes in lymphatic vessel. In 2001, it was reported that lymph drained from RA patients' joints contains high concentrations of cytokines and chemokines [1]. In addition, increased lymphatic vessel formation appears in synovial specimens from RA patients [2]. Consistent with these clinical reports, we previously found that joint specimens from TNF transgenic (Tg) mice [3], a mouse model of chronic inflammatory arthritis, have increased lymphatic vessel formation [4, 5]. By using indocyanine green near-infrared (ICG-NIR) lymphatic imaging and other outcome measures, in the past, we demonstrated that inhibition of lymphatic drainage via intraperitoneal injection VEGFR-3 neutralizing antibody increases the severity of joint inflammation [3, 4, 6–9], while stimulation of lymphatic drainage via injection of VEGF-C adenovirus at ankle joint decreases joint inflammation and tissue damage in TNF-Tg mice [10]. These findings illustrate that sufficient lymphatic drainage is favorable for the treatment of RA. Treatment targeting joint lymphatic drainage function contributes to the improvement of chronic inflammatory arthritis [11].

Currently, anti-inflammatory and reducing sequelae are main treatment in modern medicine for RA, although recombinant protein and monoclonal antibody drugs have emerged to bring hopes for millions of RA patients, those effects remain to be further followed up. TNF inhibitors etanercept, infliximab, and adalimumab alpha and other drugs could effectively reduce swelling and pain of RA patients, but there are still many patients who do not have any response at the initial treatment of TNF inhibitors, or failure in the second times of treatment, and about 10% of the patients discontinued per year. Looking for safe and effective treatment has become a major problem in the research of RA.

Du-Huo-Ji-Sheng-Tang (DHJST), a Chinese patent medicine, which is composed of radix Angelicae Pubescentis, Herba Taxilli, Radix Acanthopanacis Bidentatae, Herba Asari, Radix Gentianae Macrophyllae, Cortex Cinnamomi,* Eucommia*, Rhizoma Chuanxiong, Radix Saposhnikoviae, Radix Saposhnikoviae, liquorice, angelica, peony,* Rehmannia*, Ginseng, and poria, has been widely used for the treatment of rheumatoid arthritis (RA) in China [12]. It can improve clinical symptoms and knee function of patients [13]. But the mechanism deserves further research. Our preliminary screening identified DHJST stimulate lymphatic vessel growth of transgenic zebrafish. Those results suggested that DHJST has good effect on lymphatic vascular system. Our hypothesis is that DHJST attenuate inflammation severity of RA through promoting lymphatic drainage function. Thus, the aim of this study is to investigate whether DHJST could attenuate inflammation and promote lymphatic drainage function of TNF-Tg mice.

## 2. Experimental

### 2.1. Animals

The 3647 lines of TNF transgenic (TNF-Tg) mice were kindly provided by Dr. G. Kollias (Institute of Immunology, Alexander Fleming Biomedical Sciences Research Center, Vari, Greece) and were backcrossed with C57BL/6 mice to be maintained as heterozygotes; therefore, nontransgenic littermates are used as aged-matched wild type (WT) controls. This line of TNF-Tg mice carries one copy of the human TNF transgene and develops a disease that closely resembles RA, which is characterized by spontaneous, chronic, progressive inflammatory erosive joint disease [14]. TNF-Tg mice have normal ankle joints when they are 1 month old; they develop mild ankle joint inflammation and bone erosion at 3 months old, which become more severe at 5 months old or older [15, 16]. Three-month-old TNF-Tg mice (mild ankle joint inflammation and bone erosion) and WT littermates were used.

The transgenic zebrafish line (fli1:egfp; gata1:DsRed), in which endothelial cells express eGFP and blood cells express DsRed [17], was kindly provided by Simon Ming Yuen Lee (Institute of Chinese Medical Sciences, Macau). It was maintained in a controlled environment described in the Zebrafish Handbook [18]. Embryos were generated by natural pairwise mating, when the fish were 3–12 months old and were raised in embryo water (13.7 mM NaCl; 540 *μ*M KCl; pH 7.4; 25 *μ*M Na_2_HPO_4_; 44 *μ*M KH_2_PO_4_; 300 *μ*M CaCl_2_; 100 *μ*M MgSO_4_; 420 *μ*M NaHCO_3_; pH 7.4).

All animal procedures were followed by the Guiding Principles for the Care and Use of Laboratory Animals of National Science and Technology Committee of China.

### 2.2. Indocyanine Green Near-Infrared (ICG-NIR) Lymphatic Imaging

ICG-NIR lymphatic imaging was performed by using Fluobeam 800 imaging system (United States) according to the method previously described [6, 15, 19–21]. Indocyanine green (Acorn) solution (0.1 *μ*g/mL, 10 *μ*L) was injected intradermally into the mouse footpad using a 30-gauge needle, after removing fur from legs with hair removal lotion. Under an infrared laser, the dynamics of ICG fluorescence over the entire leg was visualized. ICG fluorescence at the whole leg was recorded for 1 hour immediately after ICG injection and again for 5 minutes at 24 hours after ICG injection. Sequential images were analyzed for the ICG intensity of the injection site and collecting lymphatic vessels efferent ankle joint using Image J software to quantify (1) % clearance, which is an assessment of ICG washout through the lymphatics and is quantified as the percent difference of ICG signal intensity between the two ICG-NIR images from the ROI of the injection site at 1 hour after ICG injection and 24 hours later and (2) lymphatic pulse, which is the ICG pulses that pass the region of interest (ROI) of the draining lymphatic vessel afferent to PLN within 500 seconds, as we described previously [6, 15, 19–23].

### 2.3. Plant Material and Preparation

Herbs in DHJST (Table 1), provided by Shanghai Huayu Chinese Herbs Co. Ltd., China, were accredited by a pharmacognosist according to standard protocols, prepared by Longhua Hospital affiliated to Shanghai University of Traditional Chinese Medicine. The drugs were extracted according to standard methods of Chinese Pharmacopoeia (China Pharmacopoeia and Committee, 2000). The Ginseng was soaked in 12 times the volume of water for 40 mins and boiled for 40 mins, the drug solution was filtered, and the filter residue was boiled in 8 times the volume of water for another 40 mins and the solution was filtered again. Both filtrates of Ginseng were mixed to get decoction 1. The remaining 13 crude drugs except Cortex Cinnamomi were soaked in 12 times the volume of water for 40 mins and boiled for 40 mins (Cortex Cinnamomi was added at 35 mins), the drug solution was filtered, and the filter residue was boiled in 8 times volume of water for another 40 mins and the solution was filtered again. Both filtrates of the remaining 13 crude drugs were mixed to get decoction 2. Decoction 1 and decoction 2 were mixed and concentrated to 77 mL under reduced pressure, the concentration of Du-Huo-Ji-Sheng-Tang for 1.2 kg/L, and then stored at −20°C until use.

DHJST samples were diluted with 4 times the water. The standard compounds of 0.125 mg ferulic acid and 2 × 10^−3^ mg osthole were mixed and dissolved in 1 mL of methanol. The standard compounds of 1.7 mg gentiopicroside and 0.7 mg paeoniflorin were mixed and dissolved in 1 mL of methanol. All the above 4 kinds of standard compounds were purchased from the National Institute for Food and Drug Control (Beijing, China). Methanol (HPLC grade, Merck KGaA, Darmstadt, Germany) and deionized water purified using the Milli-Q Reagent Water System (Millipore, Bedford, MA, USA) were used for HPLC analysis. All final solutions were passed through a 0.22 *μ*m membrane prior to use. An aliquot of 10 *μ*L of each sample solution was injected into the HPLC system for analysis.

The high-performance liquid chromatography equipment was an Agilent 1260 series system consisting of a G1322A degasser, a G1311A quaternary pump, a G1311A autosampler, a G1316A column temperature controller, and a G1315B DAD detector (Palo Alto, CA, USA). A Waters XBridge C18 column (250 mm × 4.6 mm i.d., 5 *μ*m) was used and maintained at 30°C. The mobile phase was 0.1% formic acid aqueous solution (A) and methanol (B) with a gradient program as follows: 0~125 min, linear gradient 10→15% B; 125~185 min, linear gradient 15→21% B; 185~250 min, 21% B; 250~450 min, linear gradient 21→44% B; 450~480 min, 44% B; 480~510 min, linear gradient 44→70% B; 510~530 min, 70% B and the postrun (10 min) at a flow-rate of 0.5 mL/min. Monitoring was performed at 325 nm and 274 nm. Overlaid chromatograms indicate that ferulic acid, gentiopicroside, paeoniflorin and osthole existed in DHJST (Appendices 1 and 2 of the Supplementary Material (see Supplementary Material available online at http://dx.doi.org/10.1155/2016/7067691)). Only samples that were confirmed by HPLC to meet the pharmacopoeial specifications were used in the present study.

### 2.4. Treatment

Eight 3-month-old TNF-Tg mice were given Du-Huo-Ji-Sheng-Tang (12 g/kg) or same volume physiological saline by gavage once every day for 12 weeks. Eight 3-month-old WT littermates that accepted same volume physiological saline were used as negative control. At the twelfth week, all mice were subjected to NIR-ICG imaging again. After that, ankle joints were harvested for histology staining and analysis.

Healthy zebrafish embryos were picked out at 48 hours after fecundation (hpf) and were distributed into a 12-well microplate with 10 fishes per well. Following this, the embryos were pretreated with the 30 *μ*M VEGFR-3 kinase inhibitor (MAZ51, Calbiochem, La Jolla, CA, cat. #676492, lot. #D00152431) for 6 hrs. The medium was then replaced with different concentrations (10–100 *μ*g/mL) of DHJST for 48 hrs. Embryos treated with 0.2% DMSO served as a vehicle control and were equivalent to not treatment. Each group had more than 9-10 fishes. The effect of DHJST on lymphangiogenesis was quantified by measuring the length of lymphatic thoracic duct at 10 somites of the trunk region, spanning from somite boundary 7 or 8 to 18 of each zebrafish. The photos were taken under Confocal Fluorescence Imaging Microscope (Leica TCS-SP5, Germany).

### 2.5. Histology

Ankle joints were fixed in 10% phosphate-buffered formalin at room temperature for 48 hours, decalcified in 10% EDTA for 21 days, and embedded in paraffin. A total of 30 consecutive sections from one ankle joint were collected and at least 4 *μ*m consecutive sections were cut and mounted on common slides and were collected and divided into 3 levels. Each level was 40 *μ*m from the previous level. One section from each of the 3 levels was assessed for hematoxylin and eosin (H&E), Alcian blue/orange G (ABHO) staining or tartrate resistant acid phosphatase (TRAP) staining, and histomorphometry analysis. The inflammatory area and bone area were analyzed by using HE-stained sections, cartilage area at ankle joint was measured on ABHO-stained sections, and TRAP positive area expressed as a % of the total tissue area was measured on TRAP-stained sections. The data are presented as the mean from 3 levels cut from each joint sample.

### 2.6. Immunohistochemistry

Double immunofluorescence staining was performed on paraffin sections of ankle joints as described previously [24, 25]. Briefly, after antigen retrieval with 20 *μ*g/mL proteinase K, sections were blocked in 5% BSA-PBST for 30 min and then incubated with hamster monoclonal anti-mouse podoplanin (Abcam Inc., Cambridge, MA; cat. #ab11936; clone: 8.1.1, 1 : 1,000) and rabbit anti-mouse lymphatic vessel endothelial hyaluronan receptor 1 (LYVE-1, Abcam Inc., Cambridge, MA, USA; cat. #ab14917; lot. #GR126653-4, 1 : 1,000) in 1% BSA-PBST at 4°C overnight. Secondary antibodies, Alexa Fluor 546-goat-anti-hamster (Invitrogen-Molecular Probes, Eugene, OR; cat. #A21111, 1 : 400), and Alexa 488-conjugated goat against rabbit secondary antibody (Molecular Probes, Eugene, OR, USA; cat. #A11001; lot. #99E2-1, 1 : 400) were used to incubate sections for 2 hours after extensive washing. After mounting, the double immunofluorescence stained slides were scanned using an Olympus VS-110 whole-slide imaging system and analyzed using Olympus VS-110 software version 2.3.

We outlined regions of interest (ROIs), from the distal of tibia to the distal of talus, which included articular capsules, adjacent soft tissues, and articular cartilage and bones. The total tissue area of the ROI and the area and numbers of lymphatic vessels within the entire ROI were determined automatically with Olympus VS-110 software. The total tissue area ranged from 3.5 to 3.508 mm^2^, and the area of lymphatic vessels was calculated by dividing the lymphatic area by the total tissue area. Podoplanin+/LYVE-1+ vessels were defined as lymphatic vessels according to published literature [26–30].

### 2.7. Statistical Analysis

Data were expressed as means ± standard deviation. Statistical analyses were performed using SPSS 18.0 software. One-way ANOVA test followed by Bonferroni posttest was used for multiple group comparisons. Statistically significant differences were considered when *p* < 0.05.

## 3. Results

### 3.1. DHJST Decreases Severity of Arthritis in TNF-Tg Mice

**Figure 1:**
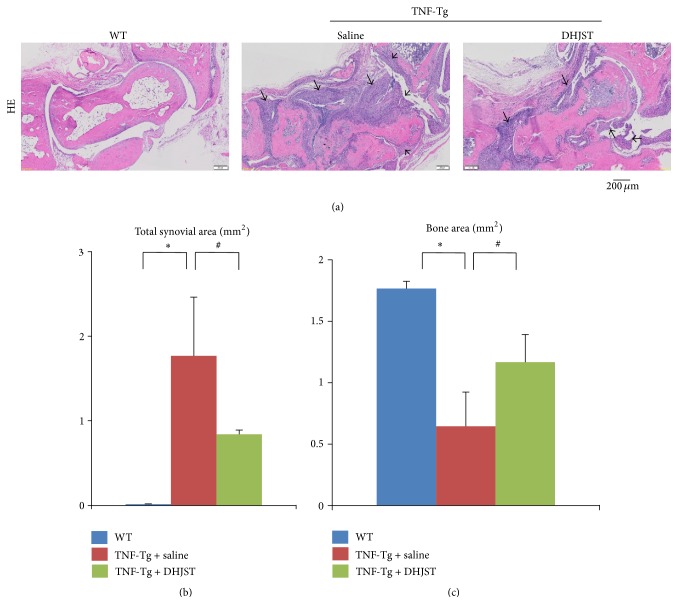
Du-Huo-Ji-Sheng-Tang reduces inflammation and bone erosion in TNF-Tg mice. Three-month-old TNF-Tg mice were treated with Du-Huo-Ji-Sheng-Tang (12 g/kg/gavage, daily ×12 weeks) or saline. Ankle joints were harvested and subjected to histologic analysis. WT mice were included as control. (a) Representative HE-stained sections show decreased inflammation and bone loss in the Du-Huo-Ji-Sheng-Tang-treated joints. Bar, 200 *μ*m, black arrow indicates inflammatory synovial tissue. Quantitation of bone area (b) and inflammatory synovial area (c). Values are the mean ± SD of 6–8 legs per group. ^*∗*^ *p* < 0.05 versus WT; ^#^ *p* < 0.05 versus TNF-Tg + saline.

**Figure 2:**
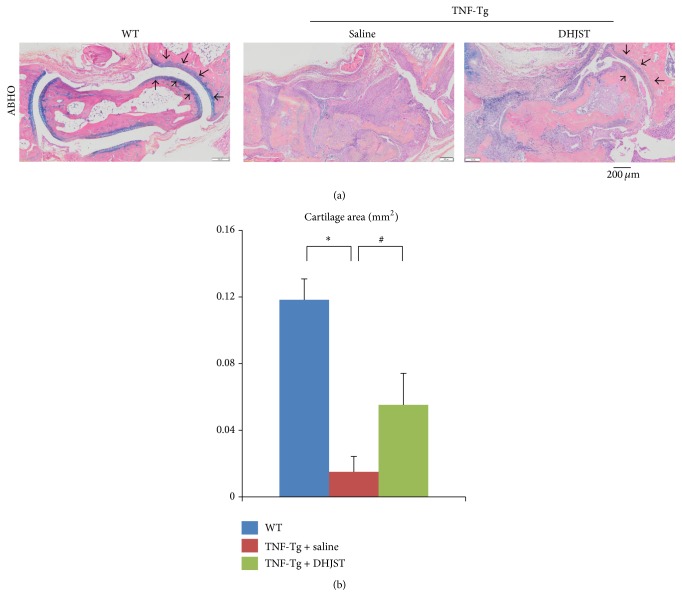
Du-Huo-Ji-Sheng-Tang reduces cartilage erosion in TNF-Tg mice. (a) Representative ABHO-stained sections show loss of cartilage at ankle joint of saline treated TNF-Tg mice and reduced cartilage erosion in the Du-Huo-Ji-Sheng-Tang-treated joints. Bar, 200 *μ*m, Alcian blue positive staining (black arrow) indicates cartilage at ankle joint. Quantitation of cartilage area (b). Values are the mean ± SD of 6–8 legs per group. ^*∗*^ *p* < 0.05 versus WT; ^#^ *p* < 0.05 versus TNF-Tg + saline.

**Figure 3:**
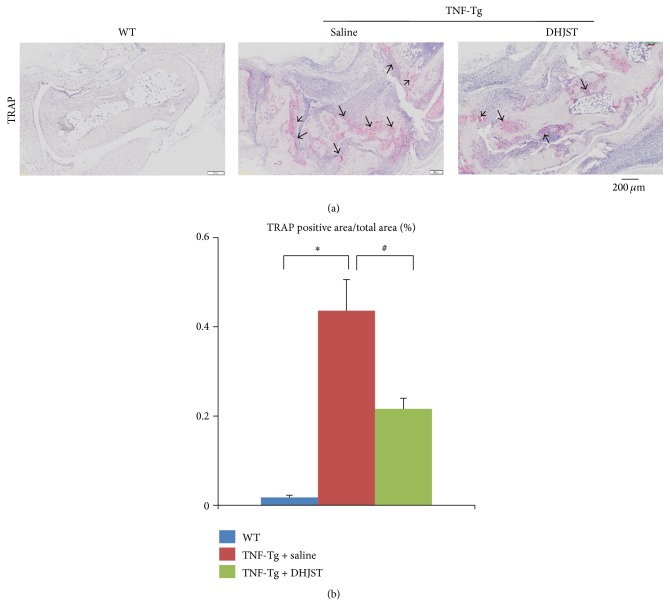
Du-Huo-Ji-Sheng-Tang reduces TRAP+ osteoclast in TNF-Tg mice. (a) Representative TRAP-stained sections show decreased TRAP+ osteoclast number in the Du-Huo-Ji-Sheng-Tang-treated joints. Bar, 200 *μ*m, arrow indicates TRAP+ osteoclast. Quantitation of TRAP+ osteoclast area (b). Values are the mean ± SD of 6–8 legs per group. ^*∗*^ *p* < 0.05 versus WT; ^#^ *p* < 0.05 versus TNF-Tg + saline.

To investigate the effect of DHJST on arthritis, TNF-Tg mice were given DHJST by gavage daily for 12 weeks. We quantified synovial inflammation and bone erosion in HE staining sections of ankle joints, cartilage damage in ABHO staining sections, and osteoclast invasion in TRAP staining section. Synovial volume in ankle joint of WT mice is 0.015 ± 0.0044 mm^2^, while severely increased synovial inflammation volume was observed in saline treated TNF-Tg mice (1.767 ± 0.69 mm^2^). Impressively, DHJST treatment significantly reduced inflammatory synovial volume of TNF-Tg mice, versus saline treated control (Figures 1(a) and 1(b)). In addition, significantly reduced bone area was observed in saline treated TNF-Tg mice, versus WT littermates. However DHJST treatment restored the bone volume of TNF-Tg mice, compared with saline treated group (Figures 1(a) and 1(c)). Besides that, the cartilage area is also significantly reduced in saline treated TNF-Tg mice versus WT mice. Compared with saline treated TNF-Tg mice, DHJST significantly increased cartilage area of TNF-Tg mice (Figures 2(a) and 2(b)). Furthermore, severely increased osteoclast area was observed in ankle joints of saline treated TNF-Tg mice, which was significantly reduced by DHJST treatment (Figures 3(a) and 3(b)).

### 3.2. DHJST Promotes Lymphatic Drainage Function in TNF-Tg Mice

**Figure 4:**
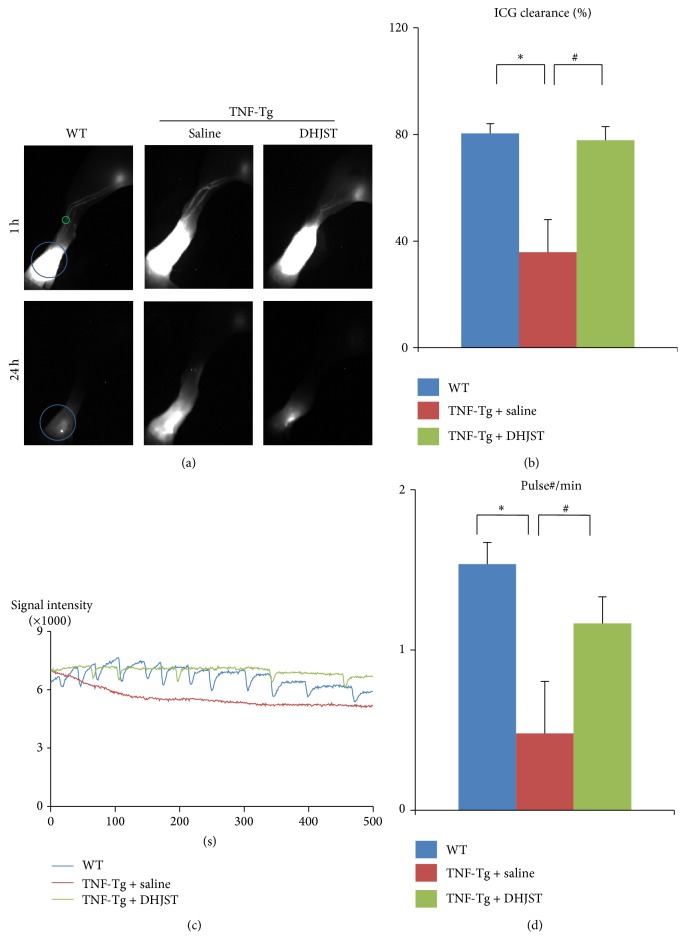
Impaired lymphatic function of TNF-Tg mice was rescued by Du-Huo-Ji-Sheng-Tang. Three-month-old TNF-Tg mice were treated with Du-Huo-Ji-Sheng-Tang or saline for 12 weeks and were subjected to ICG-NIR imagining. (a) Representative ICG images show that Du-Huo-Ji-Sheng-Tang increased ICG removal from ankle area. (b) Quantitation of % ICG clearance. Values are mean ± SD of 8–18 legs. (c) Lymphatic pulses were measured at a region of interest. Histogram shows that Du-Huo-Ji-Sheng-Tang restored lymphatic pulses in TNF-Tg mice. (d) Quantitation of lymphatic pulses/min. Values are mean ± SD of 7–18 legs from 4–9 mice. ^*∗*^ *p* < 0.05 versus WT mice, ^#^ *p* < 0.05 versus saline treated TNF-Tg group.

Consistent with previous study [3, 10, 20, 22, 23], lymphatic drainage function (including clearance and pulse) was impaired in TNF-Tg mice. But DHJST increased the ICG clearance at the footpad and the pulse of efferent lymphatic vessel between popliteal lymph node and footpad of TNF-Tg mice (Figures 4(a)–4(d)).

### 3.3. DHJST Promotes Lymphangiogenesis in Ankle Joint of TNF-Tg Mice

**Figure 5:**
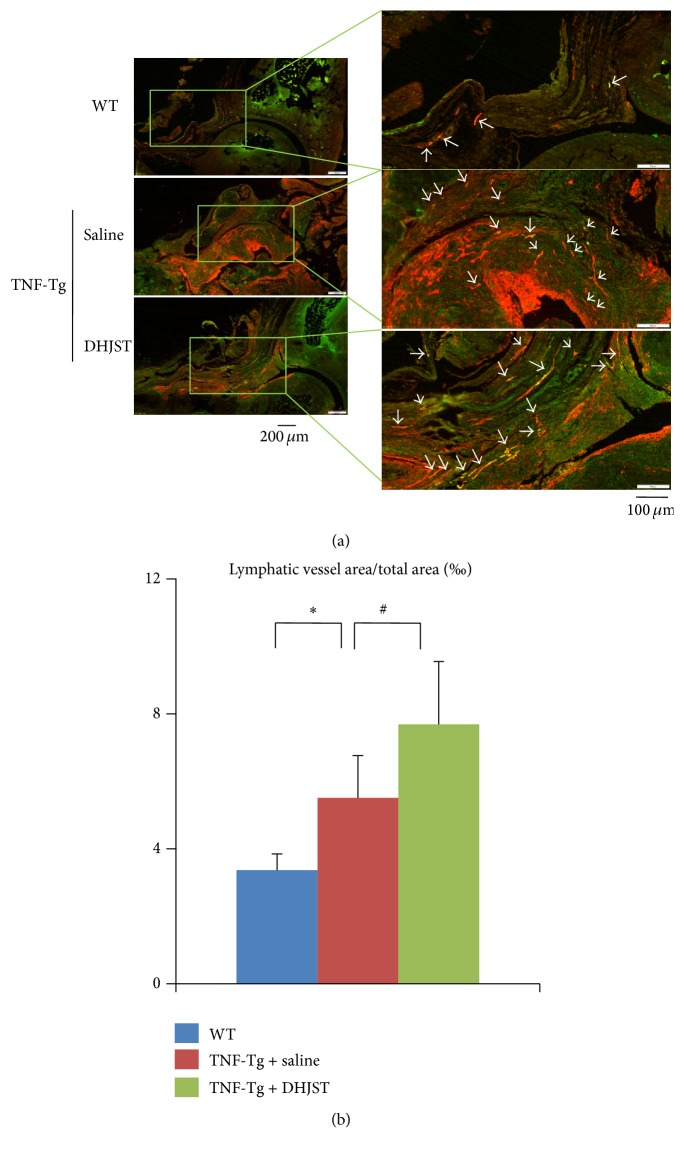
DHJST increases lymphangiogenesis in ankle joints of TNF-Tg mice. (a) Three-month DHJST or saline treatment, ankle joints were harvested and subjected to double immunofluorescence staining with anti-LYVE-1 and anti-podoplanin antibodies. Representative LYVE-1 (green) and podoplanin (red) stained ankle sections show that LYVE-1+/podoplanin+ lymphatic vessels (white arrows) are present at synovium and soft tissue surrounding the ankle joints of WT and TNF-Tg mice. (b) Quantitation of LYVE-1+/podoplanin+ lymphatic vessel area inside the areas of ankle joint. Values are the means ± SD of 5-6 legs per group. ^*∗*^ *p* < 0.05 versus WT group, ^#^ *p* < 0.05 versus saline treated TNF-Tg group.

Previously we reported that TNF-Tg mice develop ankle synovitis and significantly increased numbers of LYVE-1+ lymphatic vessels [4]. To investigate the effect of DHJST on lymphatic formation at ankle joint of TNF-Tg mice and avoid nonspecific staining, we performed double immunofluorescence staining with anti-LYVE-1 and anti-podoplanin antibodies to visualize lymphatic vessel endothelial cells. We found a similar increase of podoplanin+/LYVE-1+ lymphatic vessels in saline treated TNF-Tg mice, compared with WT littermates. Interestingly, we observed that DHJST treatment significantly increased podoplanin+/LYVE-1+ lymphatic vessels of TNF-Tg mice (Figures 5(a) and 5(b)).

### 3.4. DHJST Promotes Lymphangiogenesis in Zebrafish

**Figure 6:**
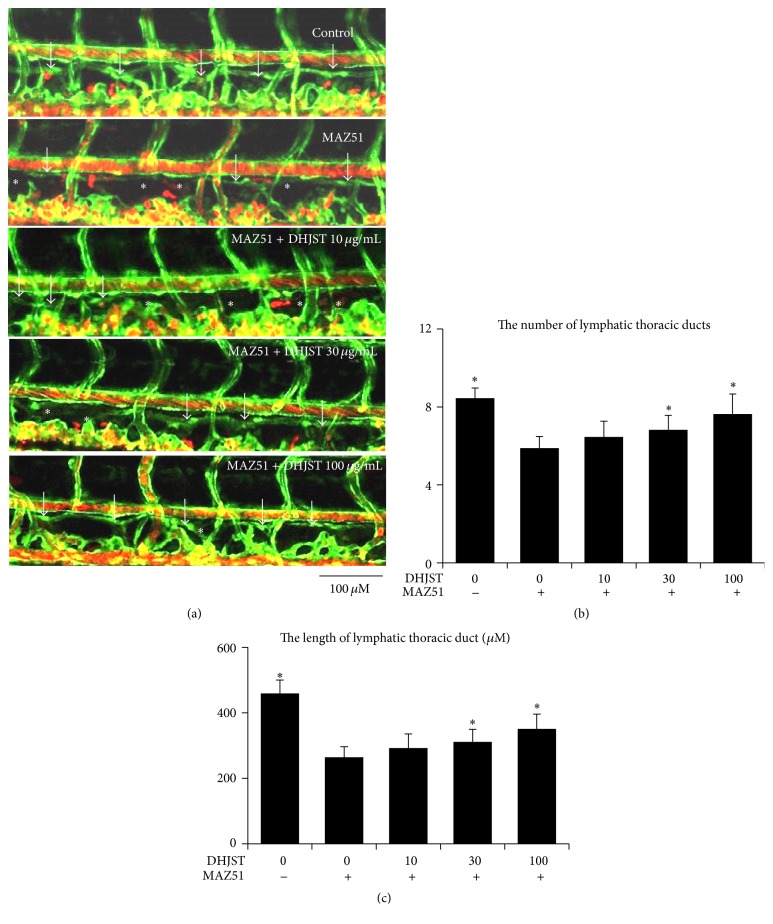
Impaired lymphatic thoracic duct formation induced by VEGFR-3 kinase inhibitor (MAZ51) was rescued by Du-Huo-Ji-Sheng-Tang in dose dependent manner. Two dpf zebrafish (fli1:egfp; gata1:DsRed) were treated with 30 *μ*M MAZ51 for 6 hours and then changed to be treated with different doses of Du-Huo-Ji-Sheng-Tang (10–100 *μ*g/mL) for 48 hours. Embryos treated with 0.2% DMSO served as a vehicle control. (a) Representative confocal images show that Du-Huo-Ji-Sheng-Tang increased lymphatic thoracic duct formation of zeberafish, white arrow indicates lymphatic thoracic duct, and white star indicates lack of lymphatic vessel. (b) Quantitation of the number of lymphatic thoracic ducts. (c) Quantitation of the length of lymphatic thoracic duct. Values are mean ± SD of 9–11 zebrafishes. ^*∗*^ *p* < 0.05 versus MAZ51.

In order to determine the effect of DHJST on lymphatic vessel, we used zebrafish screening system. In zebrafish, by 5 days after fertilization, the lymphatic thoracic duct (TD) is fully developed [21]. Thus we added VEGFR-3 specific inhibitor for 6 hours 48 hpf and then changed the medium with different doses of DHJST (10, 30, and 100 *μ*g/mL) for 48 hours. In our study, we found that MAZ51 severely impaired the number and length of TD formation, but DHJST treatment for 48 hours increased the number and length of TD formation in dose dependent manner (Figures 6(a)–6(c)).

## 4. Discussion

The objective of this study was to assess the effects of Du-Huo-Ji-Sheng-Tang on the inflammatory severity at ankle joints of TNF-Tg mice. These mice were chosen as a rheumatoid arthritis animal model because this transgenic (Tg) mouse overexpresses human TNF-alpha and develops erosive polyarthritis with many characteristics observed in rheumatoid arthritis patients, which has already been widely used for dissecting the molecular mechanisms of the pathogenic process and evaluating the efficacy of novel therapeutic strategies for rheumatoid arthritis [31]. In our current study, we found that DHJST reduced the inflammation severity, increased bone volume, and decreased TRAP+ osteoclast invasion of TNF-Tg mice. This result suggests that DHJST is an effective treatment on the inflammation severity of RA.

Our previous studies suggested that sufficient lymphatic drainage was essential for RA, and therapeutic approaches targeting joint lymphatic function showed promise for chronic inflammatory arthritis [11]. In order to investigate the lymphatic drainage function, our group previously established NIR-ICG lymphatic draining function imaging system [6, 15, 19–21]. Based on this imaging system, we found that DHJST could effectively promote the ICG clearance and pulse of efferent lymphatic vessel between footpad and popliteal lymph node, which suggested that DHJST was an effective drug for lymphatic drainage function. Consistent with our study, it was reported that DHJST has a good therapeutic effect on lymphedema syndrome [32].

It has been widely accepted that inflammation stimulates lymphangiogenesis as a compensatory mechanism to augment the clearance of inflammatory products [10]. We found that increased podoplanin+/LYVE-1+ lymphatics vessels in synovium of TNF-Tg mice are insufficient to be fully effective and that DHJST can rectify these deficiencies. Thus functional improvement of lymphatic vessels within the inflammatory sites and surrounding tissues is another therapeutic alternative to inflammation-induced edema and tissue damage.

Zebrafish has become a popular animal model in the fields of drug screening and has been widely used for assessment of efficacy and toxicity of any types of drugs, especially for multi-ingredient drugs, herbs, and exacts [33]. In current study, it is the first time for us to use zebrafish to determine the effect of drugs and herbs on lymphatic vessel. We used TG (gata1:DsRed; fli1:EGFP) zebrafish, in which the blood flow is visible in red (gata1:DsRed) and the lymphatic vessel and blood vessels are visible in green. Thus we could identify the green lymphatic vessel from blood vessel, which has red blood cells in the vessel. By using zebrafish we found that DHJST could accelerate lymphangiogenesis at the trunk of zebrafish after VEGFR3 inhibitor impairment. This result indicated that DHJST has good therapeutic effect on lymphatic vessel after impairment, and this may account for the good effect of DHJST on the lymphatic drainage function.

DHJST is one of Chinese patent medicines (CPMs), which is widely used for the treatment of rheumatoid arthritis (RA) [12] and osteoarthritis [34–36]. It was reported that DHJST inhibited sodium nitroprussiate-induced apoptosis [37] and promoted proliferation of chondrocytes [38–40]. Duhuo attenuated adjuvant-induced hind paw inflammation and hyperalgesia in rats [41]. Previous quality control study of DHJST suggested that this drug comprises osthole, gentiopicroside, loganic acid, and paeoniflorin [42, 43]. Osthole exhibited anti-inflammation [44, 45] and anti-bone-resorption effect [46] and stimulated osteoblast differentiation and bone formation by activation of beta-catenin-BMP signaling [47]. Gentiopicroside exhibits analgesic activities in the mice [48]. Loganic acid decreased proinflammatory cytokines in hypercholesterolemic rabbits [49]. Paeoniflorin is able to suppress inflammation in experimental arthritis by inhibiting abnormal proliferation of lymphocytes and synoviocytes and the production of proinflammatory cytokines and chemokines, nitric oxide, VEGF, and GM-CSF by synoviocytes [50, 51]. Ferulic acid, isolated from Rhizoma Chuanxiong and Dang-Gui [52] and Panax Notoginseng Saponins extracts from Ginseng, has antioxidative and anti-inflammatory effects [53, 54]. All the above study indicates that DHJST has anti-inflammatory actions, consistent with the result in current study that DHJST inhibits inflammation of TNF-Tg mice. But in our study, we firstly found that the promoting effect of DHJST on lymphatic drainage function is associated with reduced inflammation. And this improvement effect might be an important way for DHJST to attenuate inflammation of TNF-Tg mice.

## 5. Conclusion

DHJST inhibits inflammation severity and promotes lymphatic drainage function of TNF-Tg mice. DHJST is a promising medicine for RA and the mechanism is associated with lymphangiogenesis and lymphatic drainage function.

## Supplementary Material

Appendix 1. HPLC analysis of the components of Du-Huo-Ji-Sheng-Tang (DHJST) at 325 nm (a-c) and 274 nm (d-f). Characterized profile (chromatograms at 325 nm) of DHJST (a), the standard compounds of Ferulic Acid and Osthole (b), and blank (c) from three independent experiments. Characterized profile (chromatograms at 274 nm) of DHJST (d), the standard compounds of Gentiopicroside and paeoniflorin (e), and blank (f) from three independent experiments.
Appendix 2. HPLC data of thee batches of DHJST (g, h & i: chromatograms at 325 nm; j, k & l: chromatograms at 274 nm.).

## Figures and Tables

**Table 1:** Prescription of Du-Huo-Ji-Sheng-Tang (DHJST).

Latin name	Amount (g)
Radix angelicae pubescentis	9 g
Taxillus sutchuenensis (Lecomte) danser	6 g
Radix Acanthopanacis Bidentatae	6 g
*Asarum sieboldii* Miq.	6 g
Radix Gentianae Macrophyllae	6 g
Cortex Cinnamomi cassia	6 g
Sclerotium poriae cocos	6 g
*Eucommia ulmoides*	6 g
Radix ledebouriellae divaricatae	6 g
*Ligusticum chuanxiong* Hort.	6 g
*Panax ginseng* C. A. Mey.	6 g
*Glycyrrhiza uralensis* Fisch.	6 g
*Cynanchum otophyllum*	6 g
*Rehmannia glutinosa* (Gaertn.) Libosch. ex Fisch. et Mey.	6 g

## References

[1] Olszewski W. L., Pazdur J., Kubasiewicz E., Zaleska M., Cooke C. J., Miller N. E. (2001). Lymph draining from foot joints in rheumatoid arthritis provides insight into local cytokine and chemokine production and transport to lymph nodes. *Arthritis and Rheumatism*.

[2] Wauke K., Nagashima M., Ishiwata T., Asano G., Yoshino S. (2002). Expression and localization of vascular endothelial growth factor-C in rheumatoid arthritis synovial tissue. *The Journal of Rheumatology*.

[3] Zhou Q., Wood R., Schwarz E. M., Wang Y.-J., Xing L. (2010). Near-infrared lymphatic imaging demonstrates the dynamics of lymph flow and lymphangiogenesis during the acute versus chronic phases of arthritis in mice. *Arthritis and Rheumatism*.

[4] Zhang Q., Lu Y., Proulx S. T. (2007). Increased lymphangiogenesis in joints of mice with inflammatory arthritis. *Arthritis Research & Therapy*.

[5] Polzer K., Baeten D., Soleiman A. (2008). Tumour necrosis factor blockade increases lymphangiogenesis in murine and human arthritic joints. *Annals of the Rheumatic Diseases*.

[6] Proulx S. T., Kwok E., You Z. (2007). Longitudinal assessment of synovial, lymph node, and bone volumes in inflammatory arthritis in mice by in vivo magnetic resonance imaging and microfocal computed tomography. *Arthritis and Rheumatism*.

[7] Baluk P., Tammela T., Ator E. (2005). Pathogenesis of persistent lymphatic vessel hyperplasia in chronic airway inflammation. *The Journal of Clinical Investigation*.

[8] Flister M. J., Wilber A., Hall K. L. (2010). Inflammation induces lymphangiogenesis through up-regulation of VEGFR-3 mediated by NF-*κ*B and Prox1. *Blood*.

[9] Jeltsch M., Kaipainen A., Joukov V. (1997). Hyperplasia of lymphatic vessels in VEGF-C transgenic mice. *Science*.

[10] Zhou Q., Guo R., Wood R. (2011). Vascular endothelial growth factor C attenuates joint damage in chronic inflammatory arthritis by accelerating local lymphatic drainage in mice. *Arthritis and Rheumatism*.

[11] Buckland J. (2011). Experimental arthritis: targeting joint lymphatic function. *Nature Reviews Rheumatology*.

[12] Zhao J., Zha Q., Jiang M., Cao H., Lu A. (2013). Expert consensus on the treatment of rheumatoid arthritis with chinese patent medicines. *Journal of Alternative and Complementary Medicine*.

[13] Qin L. C. (2013). A randomized controlled observation on the combination of duhuojisheng decoction and 99Tc-MDP in the treatment of rheumatoid arthritis. *Journal of Practical Traditional Chinese Internal Medicine*.

[14] Keffer J., Probert L., Cazlaris H. (1991). Transgenic mice expressing human tumour necrosis factor: a predictive genetic model of arthritis. *The EMBO Journal*.

[15] Guo R., Zhou Q., Proulx S. T. (2009). Inhibition of lymphangiogenesis and lymphatic drainage via vascular endothelial growth factor receptor 3 blockade increases the severity of inflammation in a mouse model of chronic inflammatory arthritis. *Arthritis and Rheumatism*.

[16] Li P., Schwarz E. M., O'Keefe R. J. (2004). Systemic tumor necrosis factor alpha mediates an increase in peripheral CD11bhigh osteoclast precursors in tumor necrosis factor alpha-transgenic mice. *Arthritis and Rheumatism*.

[17] Omae M., Takada N., Yamamoto S., Nakajima H., Sato T. N. (2013). Identification of inter-organ vascular network: vessels bridging between organs. *PLoS ONE*.

[18] Westerfield M. (1995). *The Zebrafish Book. A Guide for the Laboratory Use of Zebrafish (Danio Rerio)*.

[19] Proulx S. T., Kwok E., You Z. (2007). MRI and quantification of draining lymph node function in inflammatory arthritis. *Annals of the New York Academy of Sciences*.

[20] Li J., Ju Y., Bouta E. M. (2013). Efficacy of B cell depletion therapy for murine joint arthritis flare is associated with increased lymphatic flow. *Arthritis and Rheumatism*.

[21] Luo Y., Chen W., Zhou H. (2011). Cryptotanshinone inhibits lymphatic endothelial cell tube formation by suppressing VEGFR-3/ERK and small GTPase pathways. *Cancer Prevention Research*.

[22] Bouta E. M., Wood R. W., Brown E. B., Rahimi H., Ritchlin C. T., Schwarz E. M. (2014). In vivo quantification of lymph viscosity and pressure in lymphatic vessels and draining lymph nodes of arthritic joints in mice. *The Journal of Physiology*.

[23] Bouta E. M., Wood R. W., Perry S. W. (2011). Measuring intranodal pressure and lymph viscosity to elucidate mechanisms of arthritic flare and therapeutic outcomes. *Annals of the New York Academy of Sciences*.

[24] Shi J. X., Liang Q. Q., Wang Y. J., Mooney R. A., Boyce B. F., Xing L. (2013). Use of a whole-slide imaging system to assess the presence and alteration of lymphatic vessels in joint sections of arthritic mice. *Biotechnic & Histochemistry*.

[25] Shi J., Liang Q., Zuscik M. (2014). Distribution and alteration of lymphatic vessels in knee joints of normal and osteoarthritic mice. *Arthritis and Rheumatology*.

[26] Bianchi R., Teijeira A., Proulx S. T. (2015). A transgenic Prox1-Cre-tdTomato reporter mouse for lymphatic vessel research. *PLoS ONE*.

[27] Wu X., Yu Z., Liu N. (2012). Comparison of approaches for microscopic imaging of skin lymphatic vessels. *Scanning*.

[28] Baluk P., McDonald D. M. (2008). Markers for microscopic imaging of lymphangiogenesis and angiogenesis. *Annals of the New York Academy of Sciences*.

[29] Loukovaara S., Gucciardo E., Repo P., Lohi J., Salven P., Lehti K. (2015). A case of abnormal lymphatic-like differentiation and endothelial progenitor cell activation in neovascularization associated with hemi-retinal vein occlusion. *Case Reports in Ophthalmology*.

[30] Kaser-Eichberger A., Schrödl F., Trost A. (2015). Topography of lymphatic markers in human iris and ciliary body. *Investigative Opthalmology & Visual Science*.

[31] Li P., Schwarz E. M. (2003). The TNF-*α* transgenic mouse model of inflammatory arthritis. *Springer Seminars in Immunopathology*.

[32] Yang X.-H., Liu H., Chai J.-H., Zhao X.-C. (2009). Observation on 27 elderly women in britain with lymphedema syndrome treated by acupuncture combined with medicine. *Chinese Acupuncture & Moxibustion*.

[33] Tian L. L., Zhu G. F. (2015). Application of zebra fishes in studies on traditional Chinese medicines. *Zhongguo Zhong Yao Za Zhi*.

[34] Lai J.-N., Chen H.-J., Chen C.-C., Lin J.-H., Hwang J.-S., Wang J.-D. (2007). Duhuo jisheng tang for treating osteoarthritis of the knee: a prospective clinical observation. *Chinese Medicine*.

[35] Ameye L. G., Chee W. S. S. (2006). Osteoarthritis and nutrition. From nutraceuticals to functional foods: a systematic review of the scientific evidence. *Arthritis Research & Therapy*.

[36] Teekachunhatean S., Kunanusorn P., Rojanasthien N. (2004). Chinese herbal recipe versus diclofenac in symptomatic treatment of osteoarthritis of the knee: a randomized controlled trial [ISRCTN70292892]. *BMC Complementary and Alternative Medicine*.

[37] Liu F., Liu G., Liang W. (2014). Duhuo Jisheng decoction treatment inhibits the sodium nitroprussiate-induced apoptosis of chondrocytes through the mitochondrial-dependent signaling pathway. *International Journal of Molecular Medicine*.

[38] Chen J. S., Li X. H., Li H. T. (2013). Effect of water extracts from duhuo jisheng decoction on expression of chondrocyte G1 phase regulator mRNA. *Zhongguo Zhongyao Zazhi*.

[39] Wu G., Chen W., Fan H. (2013). Duhuo Jisheng Decoction promotes chondrocyte proliferation through accelerated G1/S transition in osteoarthritis. *International Journal of Molecular Medicine*.

[40] Wu G., Fan H., Huang Y., Zheng C., Ye J., Liu X. (2014). Duhuo Jisheng Decoctioncontaining serum promotes proliferation of interleukin1*β*induced chondrocytes through the p16cyclin D1/CDK4Rb pathway. *Molecular Medicine Reports*.

[41] Wei F., Zou S., Young A., Dubner R., Ren K. (1999). Effects of four herbal extracts on adjuvant-induced inflammation and hyperalgesia in rats. *Journal of Alternative and Complementary Medicine*.

[42] Cao X. F., Zhou L. (2010). HPLC determination of osthole in Duhuojisheng pills. *China Practical Medicine*.

[43] Wu L., Wang A., Geng H., Tian J., Liu H. (2005). Determination of paeoniflorin in Duhuojisheng mixture by HPLC-ELSD. *China Pharmacist*.

[44] Wu S.-J. (2015). Osthole attenuates inflammatory responses and regulates the expression of inflammatory mediators in HepG2 cells grown in differentiated medium from 3T3-L1 preadipocytes. *Journal of Medicinal Food*.

[45] Wang X. L., Shang X., Cui Y., Zhao X., Zhang Y., Xie M. (2015). Osthole inhibits inflammatory cytokine release through PPARalpha/gamma-mediated mechanisms in LPS-stimulated 3T3-L1 adipocytes. *Immunopharmacology and Immunotoxicology*.

[46] Zhai Y. K., Pan Y. L., Niu Y. B. (2014). The importance of the prenyl group in the activities of osthole in enhancing bone formation and inhibiting bone resorption *in vitro*. *International Journal of Endocrinology*.

[47] Tang D.-Z., Hou W., Zhou Q. (2010). Osthole stimulates osteoblast differentiation and bone formation by activation of *β*-catenin–BMP signaling. *Journal of Bone and Mineral Research*.

[48] Chen L., Liu J.-C., Zhang X.-N. (2008). Down-regulation of NR2B receptors partially contributes to analgesic effects of Gentiopicroside in persistent inflammatory pain. *Neuropharmacology*.

[49] Sozański T., Kucharska A. Z., Szumny A. (2014). The protective effect of the *Cornus mas* fruits (cornelian cherry) on hypertriglyceridemia and atherosclerosis through PPAR*α* activation in hypercholesterolemic rabbits. *Phytomedicine*.

[50] Zheng Y.-Q., Wei W., Zhu L., Liu J.-X. (2007). Effects and mechanisms of Paeoniflorin, a bioactive glucoside from paeony root, on adjuvant arthritis in rats. *Inflammation Research*.

[51] Zhang W., Dai S.-M. (2012). Mechanisms involved in the therapeutic effects of *Paeonia lactiflora* Pallas in rheumatoid arthritis. *International Immunopharmacology*.

[52] Wang S., Gao Z., Chen X. (2008). The anticoagulant ability of ferulic acid and its applications for improving the blood compatibility of silk fibroin. *Biomedical Materials*.

[53] Zhang J.-H., Wang J.-P., Wang H.-J. (2007). Clinical study on effect of total panax notoginseng saponins on immune related inner environment imbalance in rheumatoid arthritis patients. *Chinese Journal of Integrated Traditional and Western Medicine*.

[54] Park S.-H., Kim S.-K., Shin I.-H., Kim H.-G., Choe J.-Y. (2009). Effects of AIF on knee osteoarthritis patients: double-blind, randomized placebo-controlled study. *The Korean Journal of Physiology & Pharmacology*.

