# Supplementary material for: Du-Huo-Ji-Sheng-Tang Attenuates Inflammation of TNF-Tg Mice Related to Promoting Lymphatic Drainage Function
Source: Evid Based Complement Alternat Med. 2016 Apr 27;2016:7067691. doi: 10.1155/2016/7067691 (PMC4863122; doi:10.1155/2016/7067691)
Supplement: Supplementary file 1 — Appendix 1. HPLC analysis of the components of Du-Huo-Ji-Sheng-Tang (DHJST) at 325 nm (a-c) and 274 nm (d-f). Characterized profile (chromatograms at 325 nm) of DHJST (a), the standard compounds of Ferulic Acid and Osthole (b), and blank (c) from three independent experiments. Characterized profile (chromatograms at 274 nm) of DHJST (d), the standard compounds of Gentiopicroside and paeoniflorin (e), and blank (f) from three independent experiments. Appendix 2. HPLC data of thee batches of DHJST (g, h & i: chromatograms at 325 nm; j, k & l: chromatograms at 274 nm.). [file 7067691.f1.pdf]

Appendix 1. HPLC analysis of the components of Du-Huo-Ji-Sheng-Tang (DHJST) at 325 nm (a-c) and 274 nm (d-f). Characterized profile (chromatograms at 325 nm) of DHJST (a), the standard compounds of Ferulic Acid and Osthole (b), and blank (c) from three independent experiments. Characterized profile (chromatograms at 274 nm) of DHJST (d), the standard compounds of Gentiopicroside and paeoniflorin (e), and blank (f) from three independent experiments.

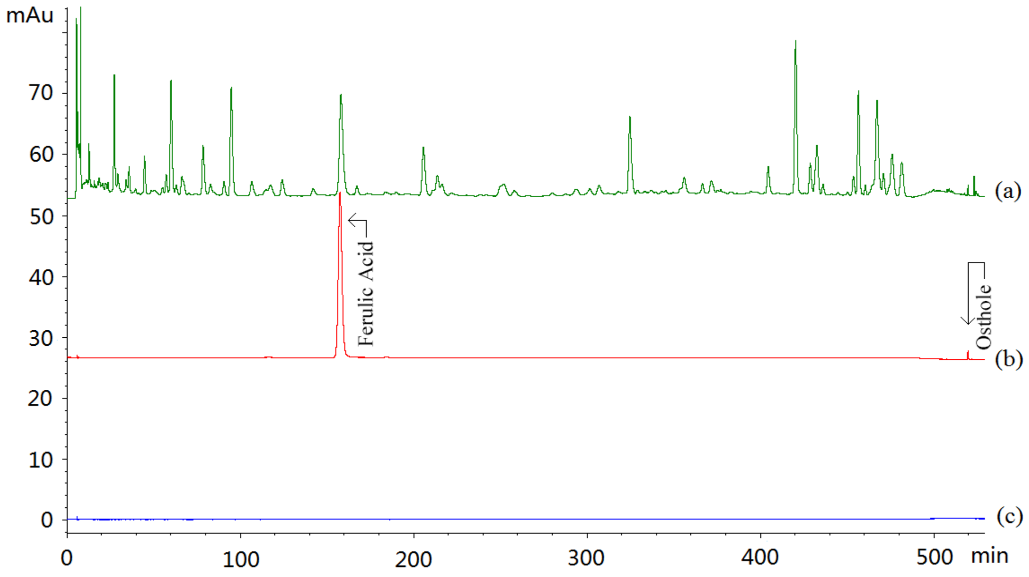

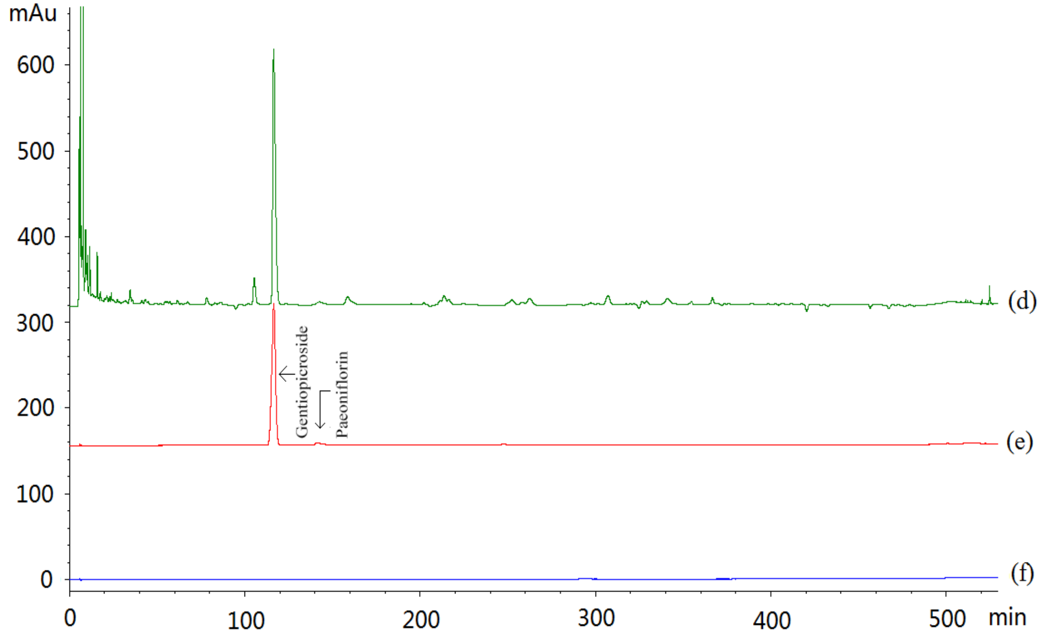

Appendix 2. HPLC data of thee batches of DHJST (g, h & i: chromatograms at 325 nm; j, k & l: chromatograms at 274 nm.).

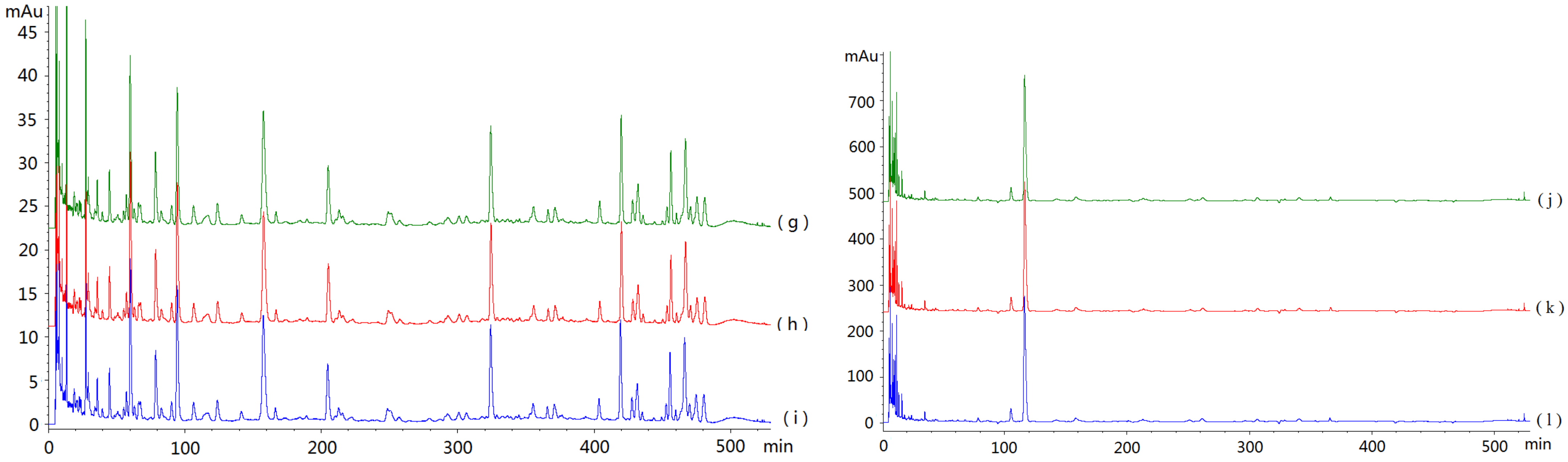

- g & j: Sample name: DHJST140822.
- h & k: Sample name: DHJST140823.
- I & l: Sample name: DHJST150127.
